# Habitat Shifts in the Pacific Saury (*Cololabis saira*) Population in the High Seas of the North Pacific Under Medium-to-Long-Term Climate Scenarios Based on Vessel Position Data and Ensemble Species Distribution Models

**DOI:** 10.3390/ani15192828

**Published:** 2025-09-28

**Authors:** Hanji Zhu, Yuyan Sun, Yang Li, Delong Xiang, Ming Gao, Famou Zhang, Jianhua Wang, Sisi Huang, Heng Zhang, Lingzhi Li

**Affiliations:** 1 Key Laboratory of Oceanic and Polar Fisheries, East China Sea Fisheries Research Institute, Chinese Academy of Fishery Sciences, Shanghai 200090, China; mikey0987@163.com (H.Z.); sunyuyan2022@163.com (Y.S.); li305338715@163.com (Y.L.); xdl17852167218@163.com (D.X.); 13155273512@163.com (M.G.); 13276209271@163.com (F.Z.); wjh20001231@163.com (J.W.); huangsisi3254@163.com (S.H.); 2 College of Navigation and Ship Engineering, Dalian Ocean University, Dalian 116023, China; 3 College of Marine Living Resource Sciences and Management, Shanghai Ocean University, Shanghai 201306, China; 4 School of Geography and Ocean Science, Nanjing University, Nanjing 210023, China; 5 School of Ecology and Environment, Anhui Normal University, Wuhu 241000, China; 6 Wenchang Innovation Research Center, East China Sea Fisheries Research Institute, Chinese Academy of Fishery Sciences, Wenchang 571343, China

**Keywords:** Pacific saury, climate change, Ensemble Species Distribution Model (ESDM), habitat suitability modeling, fishery management, North Pacific Ocean

## Abstract

**Simple Summary:**

The Pacific saury is a commercially important fish in the North Pacific Ocean that is facing threats from climate change. To understand how its habitat might change in the future, we used a novel approach that combined high-resolution satellite tracking data from fishing vessels with advanced computer models. Our highly accurate model showed that sea surface temperature and the availability of food (measured as chlorophyll-a) are the most critical factors determining where the saury live. Our projections for the future are concerning. Under a high-emission scenario, the saury’s prime habitat is predicted to shrink by over 94% by the year 2100. Furthermore, the entire population is expected to shift its center of gravity more than 400 km to the northeast. These changes could create a “mismatch” in the food web, as predators may not be able to follow the saury’s rapid move. This study provides a crucial scientific tool for international fisheries managers, like the North Pacific Fisheries Commission, to develop proactive strategies to manage this vital resource in a changing climate.

**Abstract:**

Global climate change poses a significant management challenge for vital transboundary resources like the Pacific saury (*Cololabis saira*). To address this, we developed an innovative framework that uses high-resolution Automatic Identification System (AIS) data and deep learning to define species distribution, which then informs a robust Ensemble Species Distribution Model (ESDM). The model (TSS > 0.89, AUC > 0.97) identifies sea surface temperature (SST) and chlorophyll-a (CHL) as key habitat drivers. Projections under future climate scenarios reveal two critical threats: (1) a continuous northeastward migration of the habitat’s centroid, exceeding 400 km by 2100 under a high-emission SSP5-8.5 scenario, and (2) a drastic contraction of highly suitable habitat (suitability > 0.8), shrinking by up to 94% under the high-emission SSP3-7.0 scenario. By directly linking key oceanographic features to these climate-driven risks, this study delivers an essential scientific decision-support tool for management bodies like the North Pacific Fisheries Commission (NPFC) to develop climate-adaptive strategies.

## 1. Introduction

Global climate change is profoundly reshaping the North Pacific Ocean, a region now considered a hotspot for rapid warming and acidification. Anomalous high-temperature events, such as the “The Blob” marine heatwave [1], intensify ocean stratification. This process hinders the vertical transport of deep-water nutrients to the surface, leading to a large-scale restructuring of primary productivity patterns (e.g., Chlorophyll-a concentrations) [2], which directly disrupts the foraging grounds of key species. These environmental shifts compel marine species to migrate poleward, disrupting established food webs. This impact is already evident in major commercial fisheries, with well-documented declines in iconic populations such as Pacific salmon (*Oncorhynchus* spp.) and Pacific cod (*Gadus macrocephalus*) [3,4,5].In this context, the Pacific saury (*Cololabis saira*) serves as an ideal case study. As a short-lived, migratory pelagic fish, it forms a crucial link in the food web [6], preying on zooplankton while serving as core prey for higher-trophic-level organisms such as tuna, squid, seabirds, and marine mammals. The species supports a major distant-water fishery involving multiple countries, including China, Japan, Russia, and South Korea [7,8], and is a priority for management by the North Pacific Fisheries Commission (NPFC). However, its distribution has become increasingly unpredictable, rendering traditional, experience-dependent management approaches inadequate.

To accurately forecast the saury’s future trajectory, it is essential to move beyond the limitations of traditional fishery logbooks, which often lack spatiotemporal resolution and accuracy [3]. High-resolution Automatic Identification System (AIS) data, by contrast, offering an unprecedentedly accurate source of species presence data [9,10]. While obtaining multi-national AIS data is challenging, this study utilizes a comprehensive dataset from the Chinese distant-water fleet. This fleet was selected as a robust proxy because its extensive operational range covers the main high seas fishing grounds, and its activities—representing a substantial portion of the total high-seas catch—provide a reliable reflection of the species’ overall distribution. Despite this progress, a systematic forecast of saury habitat changes under long-term climate scenarios remains a critical gap [11], a need made more urgent by the species’ recent assessment as being in an overfished state. Therefore, this study pioneers an integrated framework that combines deep learning with an Ensemble Species Distribution Model (ESDM) to quantify the key drivers of saury distribution and predict how its habitat will contract and shift [12,13]. The aim is to provide a strong scientific basis for the adaptive management and sustainable utilization of this critical resource.

## 2. Materials and Methods

### 2.1. Data Sources

#### 2.1.1. Vessel Position Data

Given that obtaining multi-national vessel position data is often challenging due to data sensitivity, this study utilized a comprehensive AIS dataset from the Chinese distant-water stick-held dip net fleet from 2019 to 2024. This technology provides unprecedented high-resolution information on the spatiotemporal distribution of fishing activities, making it a superior data source to traditional logbooks. While acknowledging the limitation of using single-nation data, this specific fleet was selected as a robust proxy for the fishery’s overall distribution because official data from the North Pacific Fisheries Commission (NPFC) confirms its dominant role. According to the NPFC Annual Summary Report, between 2019 and 2024, China’s fleet consistently accounted for approximately 30% to 37% of the total high seas catch, making it one of the top two contributors annually. This substantial contribution, combined with the fleet’s (1) extensive operational range covering the main high seas fishing grounds and its (2) advanced technological level, provides a reliable foundation for modeling the species’ habitat dynamics. The raw dataset comprised a total of 1,425,313 vessel position records. While the Chinese fleet covers major fishing grounds, excluding other nations’ data (e.g., Japan, Russia) may bias marginal habitat representation, though NPFC data suggest minimal core distribution deviation [14].

#### 2.1.2. Fishing Activity Identification

To ensure high-fidelity species presence data, we developed a dual-validation framework to identify fishing behavior from raw AIS trajectories. First, we employed a Convolutional Neural Network-Long Short-Term Memory (CNN-LSTM) model [9,10], trained on expert-labeled fishery logbooks from 10 representative vessels. The CNN-LSTM model, optimized via grid search (e.g., learning rate = 0.001, batch size = 80), achieved 96% validation accuracy by learning key operational features of the saury fleet, such as low speeds and drastic heading changes during nighttime operations (see Supplementary Materials, Section S1, for a full description of the model architecture, training process, and hyperparameter details). Second, to independently verify these predictions and enhance transparency, we applied a conservative threshold-based method grounded in established fishery knowledge, which screened for conditions such as low vessel speed (0.1–4.0 knots), significant heading changes, nighttime operations, and low wave height (<2.5 m) [15]. By integrating the results from both methods, we identified 246,700 high-confidence fishing operation points for subsequent analysis.

#### 2.1.3. Presence Data Spatial Thinning

Fishery-dependent capture data are inherently opportunistic, and their spatial distribution is often highly clustered along dominant fishing grounds and customary routes, which leads to severe sampling bias and spatial autocorrelation issues. If not addressed, these problems can distort the model’s inference of species-environment relationships [16]. To mitigate this issue and ensure a relatively balanced contribution from each environmental unit to the model construction, this study performed a spatial thinning process on the high-density fishing point data.

This research was executed using the R language, retaining only one presence record within each 1/12° × 1/12° grid cell. This grid resolution was consistent with that of the environmental data used in subsequent analyses, ensuring spatial uniformity across datasets. This procedure is not only a standardized data-cleaning process but also a critical step in satisfying the model’s assumption of statistical independence; the explicit specification of the functions and parameters ensures the complete reproducibility of the process [17], resulting in 18,911 unique presence records for subsequent model training and evaluation.

#### 2.1.4. Environmental Variables

Given Pacific saury’s ecology as a cold-water, mid-pelagic species, its distribution is closely tied to surface temperature and primary productivity [8,18,19]. Therefore, informed by a review of relevant literature and the species’ established life history [7,20], five key environmental variables were selected to construct the habitat model. The rationale for each is as follows:

Sea Surface Temperature (SST): As a stenothermal species, SST is the most critical factor governing the saury’s metabolic rate, growth, and large-scale migratory patterns [18,20].

Chlorophyll-a concentration (CHL): This variable serves as a key proxy for primary productivity, indicating the availability of zooplankton, which is the primary food source for Pacific saury.

Sea Surface Salinity (SSS): SSS is a crucial indicator of different water masses and oceanic fronts, which often define the boundaries of the saury’s preferred pelagic habitat.

Mixed Layer Depth (MLD): The MLD influences the vertical distribution of nutrients and prey, and the species are often associated with specific thermal structures in the upper water column.

Ocean Current Velocity (CV): This variable represents the dynamic stability of the oceanic environment. Saury tends to favor areas with specific current conditions for migration and foraging. For analytical convenience, CV was derived by combining northward (Vo) and eastward (Uo) sea water velocities [21].

**Figure 1:**
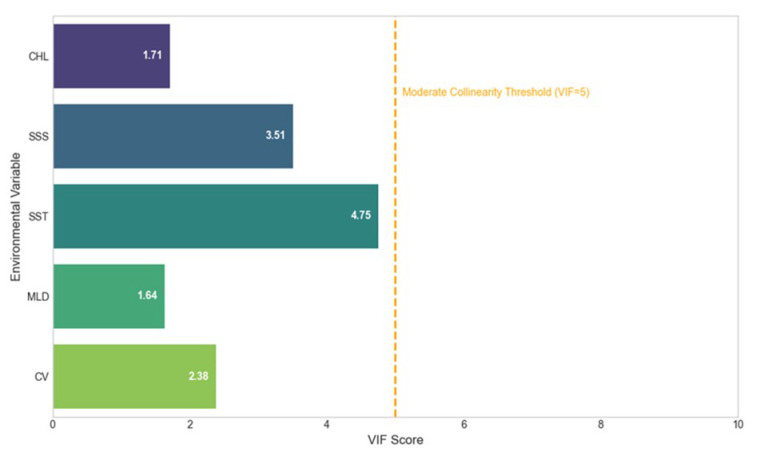
VIF Collinearity Diagnostics for Environmental Factors. The dashed orange line indicates the moderate collinearity threshold (VIF = 5).

A multicollinearity diagnostic was performed on these five variables, and all were found to be suitable for inclusion (Variance Inflation Factor, VIF < 5; see Figure 1), ensuring a robust model structure [22].

Environmental data for the current period (2019–2024) were primarily sourced from the Copernicus Marine Environment Monitoring Service (CMEMS). Data for the future periods (mid-century: 2041–2050; and end-of-century: 2091–2100) were derived from the Bio-Oracle v3.0 database (https://www.bio-oracle.org/ (accessed on 24 September 2025), which provides marine environmental variables downscaled from the Coupled Model Intercomparison Project Phase 6 (CMIP6) global climate models.

To project future habitat suitability, this study utilized climate data corresponding to four distinct Shared Socioeconomic Pathways (SSPs) provided by the Bio-Oracle database. These scenarios, developed for the Coupled Model Intercomparison Project Phase 6 (CMIP6), describe different future societal development trajectories and their corresponding greenhouse gas emission levels [23]. The four scenarios used in this study are:

SSP1-2.6: Represents a sustainable, low-emission pathway where stringent climate policies are enacted, leading to a radiative forcing of 2.6 W/m^2^ by 2100. This is often considered an optimistic or “best-case” scenario.

SSP2-4.5: A “middle-of-the-road” scenario where historical patterns of development continue, with moderate emission reductions resulting in a forcing of 4.5 W/m^2^.

SSP3-7.0: A pathway characterized by regional rivalry and slower technological development, leading to high greenhouse gas emissions and a forcing of 7.0 W/m^2^.

SSP5-8.5: A high-emission, fossil-fuel-intensive development pathway, representing a “worst-case” climate outcome with a radiative forcing of 8.5 W/m^2^.

By using these four distinct scenarios across two future periods (2050s and 2100s), we can provide a probabilistic risk assessment that captures a wide range of potential climate futures for resource managers. Detailed information for all variables is presented in the Supplementary Materials (Table S3).

#### 2.1.5. Harmonization of Environmental Data

To ensure that all predictor variables and species occurrence data were spatially aligned, all environmental layers were standardized to a uniform spatial resolution of 1/12° × 1/12°. The CHL data, which had a lower original resolution (1/4°), and all Bio-Oracle future data with an original resolution of 1/20°, were subsequently resampled using Python (version 3.10). For the current period, the monthly environmental data were averaged to generate climatological mean layers representing the 2019–2024 period. For the future data, the provided decadal mean values were used directly. All data processing was conducted within the study area defined by 135° E–180° E and 30° N–65° N at a 1/12° × 1/12° spatial resolution.

### 2.2. Ensemble Habitat Suitability Model

#### 2.2.1. Integrated Species Distribution Model Framework

To overcome the inherent biases and uncertainties of single SDM algorithms, especially when extrapolating to future climate conditions [24], this study employed an ESDM framework. This framework was implemented using the biomod2 (version 4.2.6.2) package in R (version 4.4.1), which systematically runs, evaluates, and combines eight single models. Our preliminary analysis confirmed that the predictive accuracy of the ensemble models, as measured by TSS and AUC, was significantly superior to that of any single model (see Supplementary Materials, Table S4 for detailed results). This finding is consistent with the conclusions of numerous previous studies [13,21].

During model construction, we randomly generated 50,000 background points (pseudo-absences) within the entire study area (135° E–180° E, 30° N–65° N) to avoid niche estimation bias due to insufficient background information [24]. Model performance was evaluated using five runs of random cross-validation, with the data being randomly split into an 80% training set and a 20% test set in each iteration. The primary evaluation metrics were the True Skill Statistic (TSS) and the Area Under the Curve (AUC) of the Receiver Operating Characteristic curve. TSS is insensitive to species prevalence, making it a reliable metric for assessing model performance with fishery-dependent data, which often contains sampling biases [25].

In addition to these primary metrics, we also evaluated the trade-offs between model sensitivity (correctly identifying presence points) and specificity (correctly identifying background points). To formally assess performance differences, paired *t*-tests were conducted on the metric scores from each of the five cross-validation runs. These tests revealed significant trade-offs between models; for instance, while the Classification Tree Analysis (CTA) model showed significantly higher sensitivity (*p* < 0.01), the Generalized Boosted Model (GBM) was significantly superior in specificity (*p* < 0.001). This detailed comparison informed our selection of models for the final ensemble, which balances these different performance characteristics. The full performance metrics for all single models are available in the Supplementary Materials (Table S4).

#### 2.2.2. Ensemble Model Construction and Selection

To synthesize the predictive advantages of each single model, this study constructed four ensemble models (EMmean, EMmedian, EMca, and EMwmean) for performance comparison, based on individual models that demonstrated excellent predictive performance (TSS score ≥ 0.8) during cross-validation. This strict selection criterion aims to reduce the risk of propagating model error into future projections [26]. Although our internal validation and previous research both indicate that the weighted average method (EMwmean) performs optimally [21], this study evaluated all four methods to ensure the comprehensiveness of the conclusions.

Ultimately, the selected optimal ensemble model was used to extrapolate to two future 10-year average periods: mid-century (2041–2050) and end-of-century (2091–2100). This multi-scenario, multi-period prediction approach provides environmental and fisheries managers with a probabilistic framework to assess future risks and opportunities, a critical step for developing proactive management plans [27]. Recent studies have confirmed that climate change is causing the suitable habitat of Pacific saury to migrate northward and eastward, and this study aims to quantify this trend [7].

## 3. Results

### 3.1. Model Performance

**Figure 2:**
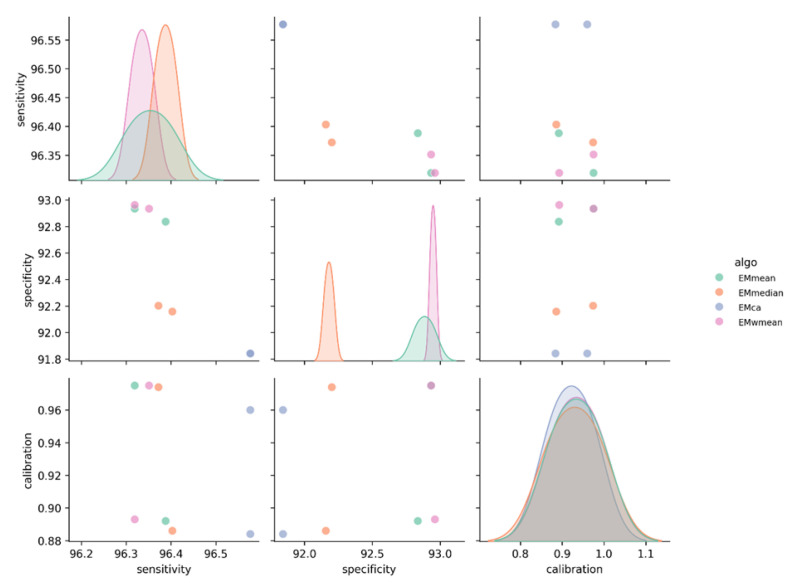
Comparative analysis among the four ensemble models.

Based on eight single models, this study constructed and evaluated the performance of four ensemble models (EMmean, EMmedian, EMca, and EMwmean) (Figure 2). The results show that all ensemble models demonstrated excellent predictive accuracy (TSS > 0.88; AUC > 0.96). Among them, the weighted mean ensemble model (EMwmean) showed the best overall performance, with a TSS score of 0.893 and an AUC of 0.975, while maintaining high levels of sensitivity (96.3%) and specificity (93.0%). Based on this evaluation, EMwmean was ultimately selected as the core model for subsequent habitat predictions.

### 3.2. Environmental Factor Contributions

**Figure 3:**
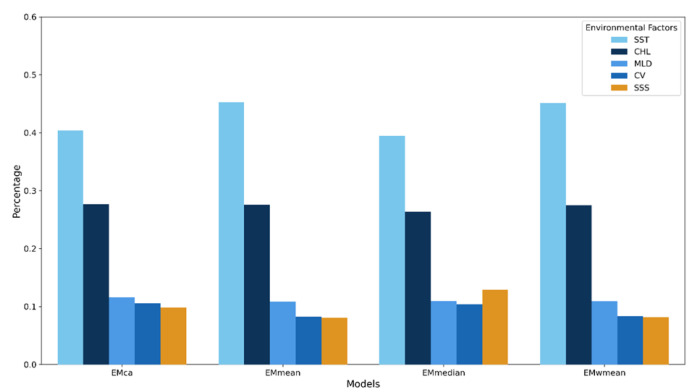
Percentage contribution of environmental factors in the ensemble models.

SST and CHL were identified as the decisive environmental factors shaping the species’ habitat patterns. This conclusion proved to be highly robust, holding true across all individual models (see Supplementary Materials, Figure S1) and ensemble models (Figure 3). In all models, the contribution rate of SST was by far the highest, ranging from 39.5% to 45.3%, making it the primary driver affecting the species’ distribution. As a key indicator of marine primary productivity, CHL was the second most important influencing factor, with its contribution rate stable at approximately 26% to 28%. The cumulative contribution of these two factors is extremely high, explaining over 65% of the variance in species distribution across all models. In comparison, the contributions of MLD, CV, and SSS were relatively small. The ranking of environmental factor importance (SST > CHL > MLD > SSS/CV) was completely consistent across all four ensemble methods.

**Figure 4:**
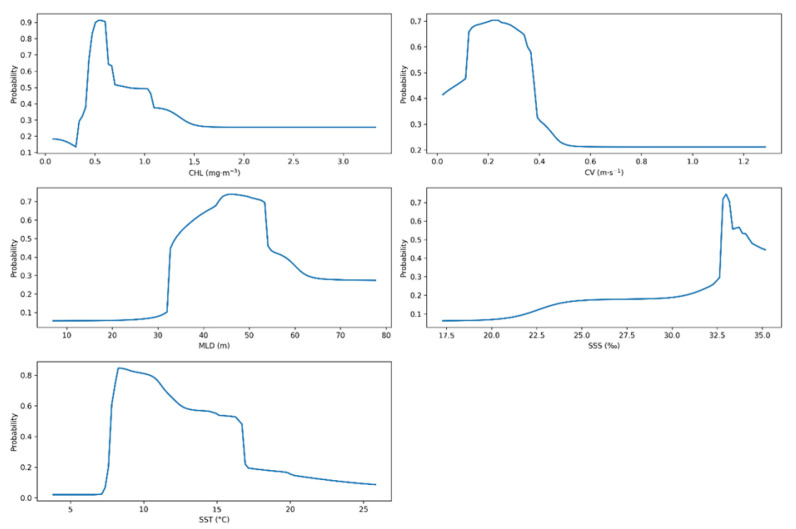
Response curves of habitat suitability for each environmental factor under the ensemble weighted model.

The response curves from the selected EMwmean model reveal the precise environmental thresholds that define the suitable ecological niche of Pacific saury (Figure 4). Habitat suitability peaked within a narrow SST range of 9–10 °C, declining rapidly in waters below 7 °C or above 15 °C. Suitability was highest at a CHL concentration of approximately 0.5 mg m^−3^. The species also showed a preference for a mixed layer depth (MLD) between 33 and 55 m, low current velocities (<0.3 m s^−1^), and Sea Surface Salinity (SSS) exceeding 32.5‰.

### 3.3. Habitat Distribution Prediction

**Figure 5:**
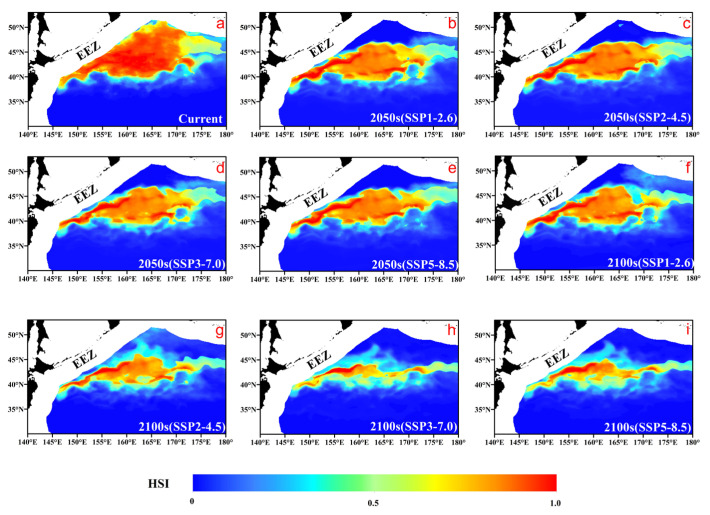
Projected changes in Pacific saury habitat suitability (HSI) for (**a**) the current period; the 2050s under scenarios (**b**) SSP1-2.6, (**c**) SSP2-4.5, (**d**) SSP3-7.0, and (**e**) SSP5-8.5; and the 2100s under scenarios (**f**) SSP1-2.6, (**g**) SSP2-4.5, (**h**) SSP3-7.0, and (**i**) SSP5-8.5.

In terms of area (Figure 5 and Table 1), projections indicate a decline in both the quality and area of the species’ habitat, with the main trends being a reduction in highly suitable habitats, an expansion of unsuitable areas, and an overall distribution shift to the north and east. The decline in habitat quality is concentrated in the changes to the high-suitability areas (HSI 0.8–1.0). Under the high-emission scenario (SSP3-7.0), this highly suitable area is projected to decrease from the current 1.2 million km^2^ to just 67,000 km^2^ by the end of the century—a reduction of 94%, representing a substantial decrease in the availability of highly suitable habitat. Concurrently, the unsuitable area (HSI 0–0.4) is projected to expand by 12.1%, while the moderately suitable area (HSI 0.6–0.8), which acts as a buffer, will also decrease by nearly 40%.

**Figure 6:**
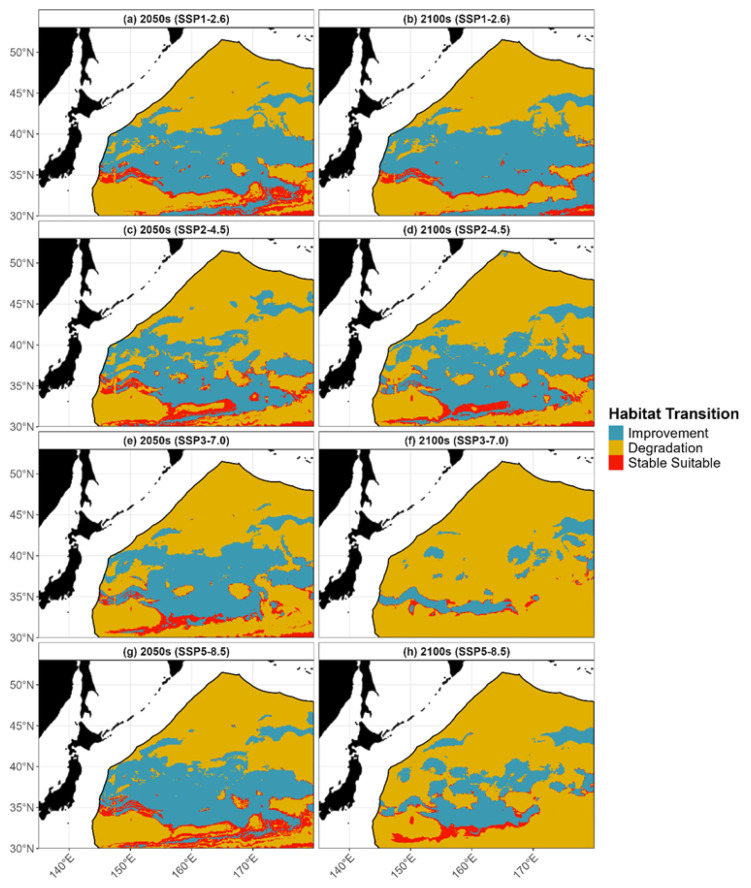
Habitat transition maps for Pacific saury under four future climate scenarios. The subfigures show transitions for the (**a**) 2050s and (**b**) 2100s under SSP1-2.6; (**c**) 2050s and (**d**) 2100s under SSP2-4.5; (**e**) 2050s and (**f**) 2100s under SSP3-7.0; and (**g**) 2050s and (**h**) 2100s under SSP5-8.5. Blue, orange, and red areas represent zones of habitat improvement, degradation, and stability, respectively, relative to the current distribution.

According to the simulations of future habitat transitions, three trends were observed: degradation, improvement, and stability, with their relative areas showing clear differences across different time periods and emission scenarios (Figure 6; see Supplementary Materials, Table S5, for detailed data). By mid-century (the 2050s), the areas of habitat degradation and improvement were relatively balanced. For example, under the low-emission SSP1-2.6 scenario, the area of improvement was approximately 2.32 million km^2^, comparable to the 2.82 million km^2^ degradation area. However, by the end of the century (the 2100s), the degradation trend became dominant. Under the high-emission SSP3-7.0 scenario, the degradation area expanded to 4.95 million km^2^, while the area available for improvement shrank to less than 0.6 million km^2^. The area of the “Stable Suitable” zone is projected to decrease from approximately 0.38 million km^2^ in the 2050s to about 0.11 million km^2^ in the 2100s under the high-emission SSP3-7.0 scenario.

**Figure 7:**
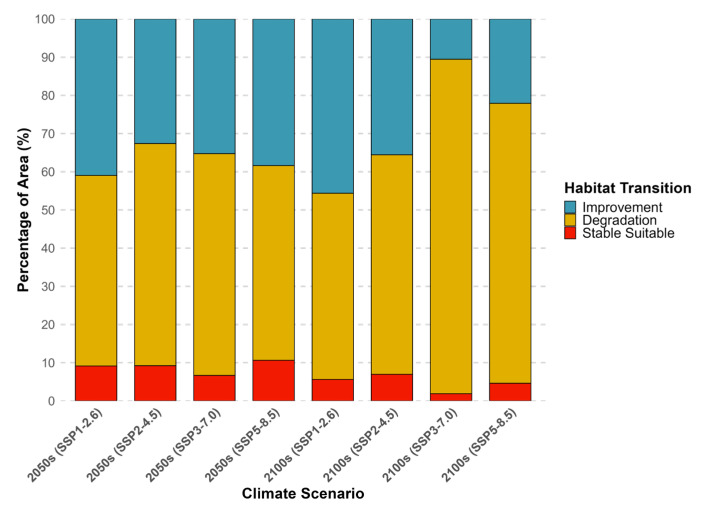
Proportional changes in Pacific saury habitat under future climate scenarios. The stacked bar chart shows the percentage of the current suitable habitat area projected to undergo Improvement (blue), Degradation (orange), or remain Stable Suitable (red) for each time period and Shared Socioeconomic Pathway (SSP).

To provide a clearer, quantitative summary of these habitat transitions, the proportional changes in area were visualized in a stacked bar chart (Figure 7). This chart highlights the dominance of the “Degradation” trend, especially by the end of the century. Under the high-emission SSP3-7.0 scenario, habitat degradation is projected to account for 87.57% of the total area, while the stable suitable habitat shrinks to just 1.88%. In contrast, under the low-emission SSP1-2.6 scenario, the area of “Improvement” remains substantial (45.63%), and the “Degradation” area is much smaller (48.77%). The precise area (km^2^) and change rate (%) for all scenarios are detailed in the Supplementary Materials (Table S5).

**Figure 8:**
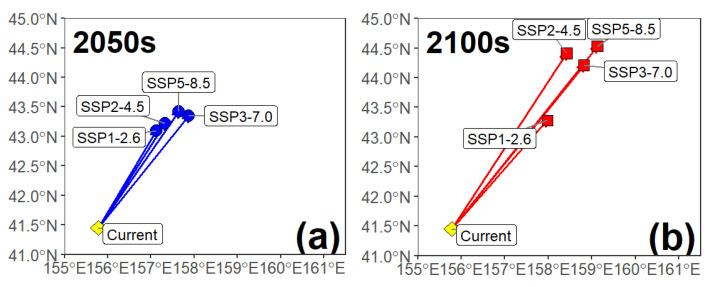
Centroid shift in Pacific saury habitat under different future climate scenarios. (**a**) Projected shift from the current period to the 2050s; (**b**) Projected shift from the current period to the 2100s.

A consistent northeastward migration trend of the Pacific saury habitat centroid from its current position was observed under all future climate scenarios (Figure 8). By mid-century (the 2050s), the centroid shifted from its current position (approx. 155.8° E, 41.5° N) to a concentrated area between 157.1° E–157.9° E and 43.1° N–43.4° N. By the end of the century (the 2100s), this migration trend continues, but the displacement magnitudes and paths diverge significantly among the different emission scenarios. Under the high-emission scenarios SSP3-7.0 (approx. 158.8° E, 44.2° N) and SSP5-8.5 (approx. 159.1° E, 44.5° N), the centroid undergoes a much larger northeastward shift. In contrast, under the low-emission SSP1-2.6 scenario, the final position of the centroid (approx. 158.0°E, 43.3°N) was much closer to the 2050s cluster. The data indicate that while the overall direction is northeastward, the final migration distance is directly related to emission intensity, with high-emission scenarios leading to a farther displacement.

## 4. Discussion

### 4.1. Model Robustness and Interpretation of Ecological Drivers

Previous studies on Pacific saury habitats have relied on fishery logbooks, remote sensing, or scientific surveys [20,28], with few utilizing high-frequency vessel position data [11]. Our study employs Automatic Identification System (AIS) data from the Chinese distant-water fleet, processed via a deep learning model to extract fishing points for an ensemble species distribution model (ESDM). While single-nation data may miss some multi-national fishery dynamics, the Chinese fleet covers major high-seas fishing grounds, and our trends align with broader studies [7]. Unlike logbooks, which suffer from limited coverage and biases, AIS data offer dynamic, high-resolution insights into fishing responses to environmental changes, improving habitat hotspot identification, which strengthens spatial management applications such as the design of dynamic protected areas [29].

The ESDM achieved robust accuracy (TSS = 0.893, AUC = 0.975, sensitivity = 96.3%, specificity = 93.0%), validating its utility as a decision-support tool for fisheries management under uncertainty [21]. Sea surface temperature (SST) and chlorophyll-a (CHL) dominate habitat distribution (39.5–45.3% and 26–28% contribution, respectively), consistent with prior research [7,20]. This indicates Pacific saury’s distribution is highly sensitive to ocean temperature and primary productivity fluctuations, guiding monitoring priorities. SST regulates metabolism and water column structure [30], while CHL reflects zooplankton availability, with saury avoiding oligotrophic and eutrophic conditions [31]. Secondary factors—mixed layer depth (MLD), ocean current velocity (CV), and sea surface salinity (SSS)—support its preference for specific thermal, current, and high-salinity (>32.5‰) environments [27,28].

As a stenothermal pelagic fish, Pacific saury thrives in a narrow thermal window of 8–12 °C [19,20]; above this range, metabolic rate and reproductive success decline [32], a sensitivity that our model confirms with a sharp suitability drop outside these optimal temperatures. Climate-driven northward shifts in the 12 °C isotherm, especially in southern marginal seas (e.g., coastal Japan), reduce traditional habitat functionality, forcing migrations. The northeastward shift in the Kuroshio–Oyashio Transition Zone creates a new ‘ecological sweet spot’ with optimal temperatures and food availability [6]. Similar shifts in herring and cod suggest global warming is redefining marine species’ ecological boundaries [4,10,33].

### 4.2. Future Spatiotemporal Habitat Shifts and Their Ecological Implications

Climate change drives community-level shifts among small pelagic fish in the Northwest Pacific, including chub mackerel (*Scomber japonicus*) and Japanese sardine (*Sardinops melanostictus*) [23,33]. Understanding Pacific saury’s response is critical for ecosystem-based management. Our study addresses a research gap in systematic, quantitative forecasts of its habitat evolution under climate scenarios by combining high-resolution AIS data with an ensemble species distribution model (ESDM) [10]. While our model is based on the Chinese fleet, our findings on the species’ northeastward shift align with broader multi-national trends [7], providing robust insights into Pacific saury’s future within the pelagic community.

Projections show three trends: a drastic contraction of highly suitable habitat (HSI > 0.8), a northeastward centroid shift, and divergent outcomes across emission scenarios. Currently, 1.2 million km^2^ of highly suitable habitat supports population reproduction, but under SSP3-7.0, this shrinks to 67,000 km^2^ by 2100—a 94% reduction, signifying a substantial decrease in prime habitat. Unsuitable areas (HSI 0–0.4) expand by 12.1% (10.89 to 12.21 million km^2^), and stable suitable areas drop from 0.38 million km^2^ (2050s) to 0.11 million km^2^ (2100s), undermining habitat resilience. This continuous shrinkage of ecological refugia risks population viability, shifting habitats from a robust, contiguous core to a fragmented, lower-quality state. By 2050s, habitat improvement (2.32 million km^2^ under SSP1-2.6) and degradation (2.82 million km^2^) balance, but by 2100, degradation dominates (5 million km^2^ under SSP3-7.0), with improvement areas shrinking to <0.6 million km^2^, reflecting high-emission pathways’ inhibitory effect on ecosystem recovery. The habitat centroid shifts from 155.8° E, 41.5° N to 159.1° E, 44.5° N by 2100 under SSP5-8.5, a >400 km displacement driven by the Kuroshio-Oyashio Transition Zone’s northward shift and thermal stress in southern habitats [6]. Pacific saury, preferring specific temperatures and zooplankton concentrations, benefits from this ‘ecological sweet spot’ with suitable conditions [7]. However, paths diverge by century’s end, reflecting climate change’s cumulative effects, with SSP1-2.6 showing a smaller shift (158.0° E, 43.3° N). Yet, geographical migration may not ensure ecological success. High-latitude regions may lack sufficient zooplankton [8], and long-distance migration could strain reproductive success. A potential trophic mismatch may occur if saury’s rapid shift outpaces predators like tuna or seabirds, though further research is needed to confirm food web impacts [32]. Such disruptions challenge single-species management, risking regional fishery stability. For the North Pacific Fisheries Commission (NPFC), these findings inform adaptive strategies to address climate-driven shifts. The >400 km centroid shift under high-emission scenarios requires re-evaluating international quota allocations, as historical distributions become outdated. The 94% habitat contraction necessitates spatial planning to optimize fishing effort, prioritizing persistent suitable areas to reduce ecological impacts. Stable suitable zones, shrinking from 0.38 million km^2^ (2050s) to 0.11 million km^2^ (2100s), suggest potential climate refugia for conservation, such as protected areas to bolster population resilience. However, key caveats limit projections: (1) niche conservatism assumes static ecological preferences, potentially overlooking adaptation to warming oceans [33]; (2) single-nation AIS data, while robust for major fishing grounds, may miss multi-national fishery dynamics; (3) omission of interspecific interactions with species like sardines and chub mackerel, whose overlapping habitats affect resource competition; and (4) uncertainties in Bio-Oracle’s downscaled climate data, particularly for small-scale oceanographic features [23,34].

While our model uses fishing locations as a proxy for species presence, it does not explicitly incorporate fishing effort as a variable. This is a common practice in large-scale habitat modeling where standardized effort data is unavailable. We acknowledge that changes in fishing technology, strategy, or intensity over time could influence the observed distribution of fishing activities. However, by using a highly standardized fleet (stick-held dip net) and a relatively short time frame for the baseline period (2019–2024), we have minimized the potential impact of such variations. Future work could benefit from integrating fine-scale effort data to further refine these projections.

Future research should prioritize mechanistic models (e.g., Individual-Based Models or Dynamic Energy Budget models) to link environmental changes [35,36], like rising SST, to population dynamics (growth, reproduction, survival). Multi-species distribution models (MSDMs) can address competitive interactions among pelagic fish [37,38,39], enhancing ecosystem-based management. Genomics and environmental DNA (eDNA) techniques should assess adaptive potential to new thermal or prey environments [40], improving model accuracy and management adaptability.

## 5. Conclusions

This study developed an innovative framework using high-resolution AIS data, deep learning, and ensemble models to predict Pacific saury habitat dynamics under climate change. Our model identified sea surface temperature (SST) and chlorophyll-a (CHL) as the core drivers shaping the species’ habitat. Projections reveal two severe threats under high-emission scenarios: a contraction of over 94% in highly suitable habitat and a northeastward migration of its centroid exceeding 400 km. This quantitative risk assessment signals profound challenges to the species’ viability and broader ecosystem stability, including potential “trophic mismatch”.

While these projections provide a critical foresight tool, they should be interpreted with caution given the methodological limitations outlined in the discussion, such as the assumption of niche conservatism and the use of single-nation fleet data. Ultimately, our work delivers an operational, science-based tool to support fisheries management bodies like the NPFC. By providing a quantitative risk assessment across different emission scenarios, these projections can directly inform the development of adaptive management strategies, including adjustments to international quota allocations, forward-looking spatial planning for fishing effort, and the identification of potential climate refugia for the species.

## Figures and Tables

**Table 1:** Changes in the area of Pacific saury habitat on the high seas in different suitability zones.

Period and Climate Scenarios	Habitat Suitability Index (HSI) Range			
	Non-Suitable Areas (0–0.4) (km^2^)	Low Suitable Area (0.4–0.6) (km^2^)	Moderately Suitable Area (0.6–0.8) (km^2^)	Highly Suitable Area (0.8–1.0) (km^2^)
Current	10,894,427	471,397	360,939	1,200,282
2050s SSP1-2.6	11,386,961	345,528	465,098	719,241
2050s SSP2-4.5	11,432,190	343,446	375,633	765,558
2050s SSP3-7.0	11,528,263	343,727	467,651	577,186
2050s SSP5-8.5	11,500,569	376,849	366,425	672,985
2100s SSP1-2.6	11,566,635	337,221	388,768	624,203
2100s SSP2-4.5	11,847,665	412,899	371,439	284,824
2100s SSP3-7.0	12,213,550	417,520	218,893	66,865
2100s SSP5-8.5	11,927,266	538,183	265,671	185,707

## Data Availability

Environmental data are publicly available from the Copernicus Marine Environment Monitoring Service (CMEMS) and the Bio-Oracle v.3.0 database. R and Python scripts used for this study, along with a sample dataset, are available on GitHub: https://github.com/saury-habitat-study/Saury-Habitat-ESDM (accessed on 24 September 2025). The raw AIS vessel position dataset is not publicly available due to data sensitivity but may be obtained from the corresponding author upon reasonable request.

## Supplementary Materials

The following supporting information can be downloaded at https://www.mdpi.com/article/10.3390/ani15192828/s1, Section S1: Detailed Description of the CNN-LSTM Model; Figure S1: Percentage contribution of environmental factors in the eight individual species distribution models; Table S1: Final Hyperparameters for the CNN-LSTM Model; Table S2: Final Model Performance Evaluation Report; Table S3: Environmental variables used in the species distribution models (SDMs) and their sources; Table S4: Summary of Single Model Performance Evaluation; Table S5: Area (km^2^) and change rate (%) of habitat transitions for Pacific saury under future climate scenarios.
