# Supplementary material for: Habitat Shifts in the Pacific Saury (Cololabis saira) Population in the High Seas of the North Pacific Under Medium-to-Long-Term Climate Scenarios Based on Vessel Position Data and Ensemble Species Distribution Models
Source: Animals (Basel). 2025 Sep 28;15(19):2828. doi: 10.3390/ani15192828 (PMC12523842; doi:10.3390/ani15192828)
Supplement: Supplementary file 1 [file animals-15-02828-s001.zip › animals-3857023-supplementary(final check).pdf]

Article

Habitat Shifts in the Pacific Saury (*Cololabis saira*) Population in the High Seas of the North Pacific Under Medium-to-Long-Term Climate Scenarios Based on Vessel Position Data and Ensemble Species Distribution Models

Hanji Zhu [1,2,†], Yuyan Sun [1,3,4,†], Yang Li [1,2], Delong Xiang [1,3], Ming Gao [1,5], Famou Zhang [1,3], Jianhua Wang [1,2], Sisi Huang [1,3], Heng Zhang [1,2,6,*] and Lingzhi Li [1,*]

1 Key Laboratory of Oceanic and Polar Fisheries, East China Sea Fisheries Research Institute, Chinese Academy of Fishery Sciences, Shanghai 200090, China; mikey0987@163.com (H.Z.); sunyuyan2022@163.com (Y.S.); li305338715@163.com (Y.L.); xdl17852167218@163.com (D.X.); 13155273512@163.com (M.G.); 13276209271@163.com (F.Z.); wjh20001231@163.com (J.W.); huangsisi3254@163.com (S.H.)
2 College of Navigation and Ship Engineering, Dalian Ocean University, Dalian 116023, China
3 College of Marine Living Resource Sciences and Management, Shanghai Ocean University, Shanghai 201306, China
4 School of Geography and Ocean Science, Nanjing University, Nanjing 210023, China
5 School of Ecology and Environment, Anhui Normal University, Wuhu 241000, China
6 Wenchang Innovation Research Center, East China Sea Fisheries Research Institute, Chinese Academy of Fishery Sciences, Wenchang 571343, China
* Correspondence: zhangziqian0601@163.com (H.Z.); lilz@ecsf.ac.cn (L.L.)
† These authors contributed equally to this work.

Supplementary Materials

Section S1: Detailed Description of the CNN-LSTM Model

(S1.1) Data Preprocessing and Model Architecture

The model for identifying fishing activity was developed using Python (v3.x) with the TensorFlow (v2.x) and Keras libraries. The raw time-series data, containing 11 features per timestamp, was standardized using StandardScaler. To structure the data for the sequential model, a sliding window of 100 timesteps was moved across each vessel's trajectory with a step of 1.

Our model integrates a Convolutional Neural Network (CNN) for spatial feature extraction with a Long Short-Term Memory (LSTM) network for capturing temporal patterns, enhanced by Squeeze-and-Excitation (SE) and Multi-Head Attention mechanisms. The specific architecture is as follows:

1. Two consecutive 1D CNN layers (64 filters, kernel size 3, 'relu' activation).
2. A Squeeze-and-Excitation (SE) block for channel-wise feature recalibration.
3. A MaxPooling1D layer (pool size 2).

4. Two consecutive LSTM layers (16 units each).
5. A Multi-Head Attention layer (2 heads) with a residual connection and Layer Normalization.
6. A GlobalAveragePooling1D layer, a Dropout layer (rate 0.5), and a final Dense output layer (8 units, 'softmax' activation).

(S1.2) Model Training, Hyperparameters, and Validation

The model was trained using a 5-fold cross-validation scheme to ensure robustness. The key hyperparameters used for training are summarized in the table below. The model was compiled with the Adam optimizer and 'categorical_crossentropy' as the loss function. An Early Stopping mechanism was employed, monitoring the validation loss with a patience of 15 epochs to prevent overfitting and restore the best model weights.

Table S1: Final Hyperparameters for the CNN-LSTM Model.

**Hyperparameter**	**Value**	**Description**
Number of Folds	5	For cross-validation
Epochs (Max)	150	Maximum number of training cycles
Batch Size	80	Number of samples per gradient update
Optimizer	Adam	Optimization algorithm
Loss Function	Categorical Crossentropy	For multi-class classification
Early Stopping Patience	15 epochs	Stops training if validation loss doesn't improve
LSTM Units	[16, 16]	Number of units in the two LSTM layers
CNN Filters	64	Number of output filters in the convolution
Attention Heads	2	Number of heads in the Multi-Head Attention layer
Dropout Rate	0.5	Fraction of the input units to drop

(S1.3) Model Performance

The performance of the final trained model was evaluated on the held-out test set from each fold. The aggregated classification report, demonstrating a weighted average F1-score of 0.96, is presented in the table below, confirming the model's high accuracy and robustness.

Table S2: Final Model Performance Evaluation Report

Metric	Precision	Recall	F1-Score	Support
Class 1	0.97	0.98	0.98	17,558
Class 2	0.93	0.93	0.93	7,390
Class 3	0.93	0.92	0.92	5,299
Class 4	0.97	0.96	0.96	28,177
Class 5	0.98	0.99	0.99	18,598
Class 6	0.95	0.92	0.93	1,336
Class 7	0.98	0.97	0.97	4,502
Class 8	0.81	0.88	0.85	5,727

Accuracy			0.96	88,587
Macro Avg	0.94	0.94	0.94	88,587
Weighted Avg	0.96	0.96	0.96	88,587

Section S2: Detailed Description of the SDMs Model

Table S3. Environmental variables used in the species distribution models (SDMs) and their sources

Variable Name	Abbreviation	Unit	Current Data	Current Original Resolution	Future Data	Temporal Coverage
Sea Surface Temperature	SST	°C	CMEMS	1/12°, Monthly	Bio-Oracle	2019–2024, 2041–2050, 2091–2100
Sea Surface Salinity	SSS	‰	CMEMS	1/12°, Monthly	Bio-Oracle	2019–2024, 2041–2050, 2091–2100
Mixed Layer Depth	MLD	m	CMEMS	1/12°, Monthly	Bio-Oracle	2019–2024, 2041–2050, 2091–2100
Eastward sea water velocity	Uo	m/s	CMEMS	1/12°, Monthly	Bio-Oracle	2019–2024, 2041–2050, 2091–2100
Northward sea water velocity	Vo	m/s	CMEMS	1/12°, Monthly	Bio-Oracle	2019–2024, 2041–2050, 2091–2100
Chlorophyll-a Concentration	CHL	mg/m^3	CMEMS	1/4°, Monthly	Bio-Oracle	2019–2024, 2041–2050, 2091–2100

Table S4. Summary of Single Model Performance Evaluation

Algo	Metric	Cutoff	Sensitivity (%)	Specificity (%)	Score (TSS/AUC)
CTA	TSS	254.5	96.32	93.44	0.898
	ROC	252	96.32	93.44	0.967
GBM	TSS	463	93.01	93.48	0.865
	ROC	475.5	92.69	93.82	0.975
ANN	TSS	204	95.24	90.31	0.856
	ROC	203.5	95.24	90.31	0.966
FDA	TSS	121	92.77	77.56	0.703
	ROC	127.5	92.15	78.24	0.927
GLM	TSS	242	94.09	77.36	0.715
	ROC	243.5	93.9	77.57	0.922
GAM	TSS	227	90.91	45.5	0.365
	ROC	227.5	90.75	45.72	0.69
MAXNET	TSS	350	92.95	87.41	0.804
	ROC	350.5	92.87	87.52	0.962
MARS	TSS	258	94.62	84.2	0.788
	ROC	264.5	94.39	84.44	0.949

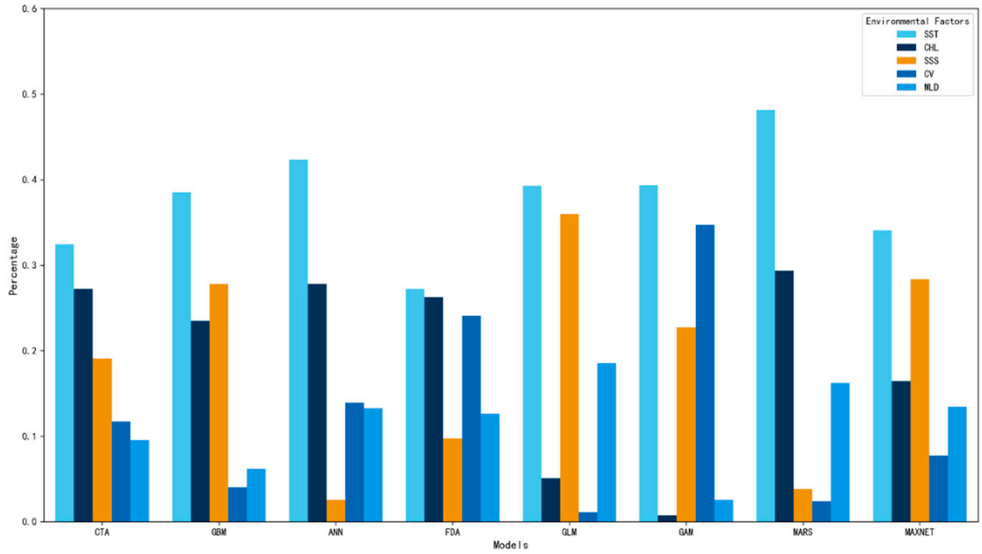

Figure S1. Percentage contribution of environmental factors in the eight individual species distribution models. This figure shows the relative importance of the five environmental variables (SST, CHL, SSS, CV, MLD) used to predict Pacific saury habitat, as estimated by each of the eight models (CTA, GBM, ANN, etc.) before ensembling.

Section S3: Detailed Description of precise area and percentage data for each category

Table S5. Area (km²) and change rate (%) of habitat transitions for Pacific saury under future climate scenarios.

Period and Climate Scenarios	Three co-existing trends					
	Improvement		Degradation		Stability	
	Area (km²)	Change rate (%)	Area (km²)	Change rate (%)	Area (km²)	Change rate (%)
2050s SSP1-2.6	2317555.7	40.96%	2823498.2	49.90%	516778.8	9.13%
2050s SSP2-4.5	1845862.9	32.62%	3290461.9	58.16%	521507.9	9.22%
2050s SSP3-7.0	1995350.1	35.27%	3285106	58.06%	377376.6	6.67%
2050s SSP5-8.5	2171904.7	38.39%	2884035.8	50.97%	601892.2	10.64%
2100s SSP1-2.6	2581470.9	45.63%	2759395.1	48.77%	316966.7	5.6%
2100s SSP2-4.5	2012463.3	35.57%	3251665	57.47%	393704.5	6.96%
2100s SSP3-7.0	596965.8	10.55%	4954653	87.57%	106213.9	1.88%
2100s SSP5-8.5	1248975.1	22.07%	4147908.3	73.31%	260949.2	4.61%
